# Current orthodontic education status in the North American region on treating patients with obstructive sleep apnea

**DOI:** 10.3389/froh.2026.1714010

**Published:** 2026-01-29

**Authors:** Jiahui Li, Chun-Hsi Chung, Wenjing Yu, Minhee Jeon, Chenshuang Li

**Affiliations:** 1 Department of Orthodontics, School of Dental Medicine, University of Pennsylvania, Philadelphia, PA, United States; 2 School of Dental Medicine, University of Pennsylvania, Philadelphia, PA, United States

**Keywords:** airway, education, obstructive sleep apnea, orthodontics, postgraduate training

## Abstract

**Objectives:**

In 2019, American Association of Orthodontists (AAO) published the white paper on obstructive sleep apnea (OSA) and orthodontics, eliciting the role of the orthodontic specialty in the management of OSA. This study aims to evaluate the current status of OSA management education in the North America region to identify areas for improvement to enhance orthodontic training and, ultimately, better patient health outcomes.

**Materials and methods:**

Multiple-choice survey links were distributed via Qualtrics to postgraduate orthodontic program directors and department chairs, requesting anonymous responses in July-September 2024.

**Results:**

The response rate was 36.5% (27 responses). 96% of respondents indicated that their program provided education on orthodontic management and treatment of OSA patients, while 74.1% reported having special protocols for managing these patients. However, teaching formats, faculty backgrounds, screening or treatment methods, and competency assessments had varying responses.

**Conclusion:**

In summary, it seems there is a lack of standardization in the post-graduate residency curriculum on OSA patient management.

## 1 Introduction

Obstructive sleep apnea (OSA), a breathing sleep disorder, is known to have neurocognitive and systemic health implications resulting from repeated episodes of partial (hypopnea) or complete (apnea) upper airway obstruction (1). These consequences vary in severity, ranging from a reduced quality of life to neurobehavioral deficits, cardiovascular morbidity, and metabolic syndrome (2). Both adults and children can be affected, with the prevalence of OSA in adults ranging from 5%–14% and in children ranging from 1%–4% (3, 4). Risk factors in adults include genetic predispositions, obesity, and craniofacial or oropharyngeal anatomic abnormalities, while the most common causes of OSA in children are hypertrophic tonsils and adenoids (4).

From an orthodontic perspective, dental features associated with OSA patients include reduced overbite, a narrow upper dental arch, a shorter dental arch, mandibular arch crowding, and Class II malocclusion (5). Although a definitive diagnosis of OSA must be made by a physician through polysomnography, these dental features highlight the important role that orthodontists play in identifying patients with risk factors for OSA (4, 6). In 2019, Behrents et al. published the American Association of Orthodontists (AAO) White Paper on OSA (4). This guideline specified that orthodontists should be familiar with signs and symptoms of adult and pediatric OSA patients, screening tools such as the STOP-Bang questionnaire and Pediatric Sleep Questionnaire, clinical examinations such as the modified Mallampati classification and Brodsky scale (4). Orthodontists should refer such patients to physicians for further evaluation and definitive diagnosis, collaborate with physicians to develop comprehensive treatment plans, and when appropriate and not contraindicated by medical management, provide adjunctive orthodontic treatment such as rapid maxillary expansion (RME) and mandibular advancement appliances for skeletal discrepancies that are indicated by the sleep medicine specialist (4).

Despite the recognized need for such expertise in the multidisciplinary treatment of OSA, there seems to be a lack of standardization in the post-graduate orthodontic curriculum on OSA patient management, and a lack of research on the evaluation of dental sleep medicine education in post-graduate orthodontic programs (7–9). For instance, when searching for literature regarding the dental education of OSA, most studies focused on undergraduate/pre-doctoral training (10–14). Only one study done in Thailand evaluated the curriculum of OSA-related training in post-graduate orthodontic residency (9, 15). With the alarming increase in the number of orthodontists marketing themselves as “airway orthodontists” and may provide unnecessary orthodontic intervention for “airway management” (16), the current study aims to evaluate the current status of OSA management education in the postgraduate orthodontic programs, and to identify areas for improvement in didactic and clinical experiences, ultimately improving patient care outcomes.

## 2 Material and methods

A request to conduct an education survey was submitted to the Institutional Review Board (IRB) of the University of Pennsylvania and was determined to be exempt (IRB#856147, approved on June 21st, 2024) as the current study does not involve any primary identifiable information of patients. The IRB exemption status was included in the survey invitation email as follows: “*The current project is exempted from the University of Pennsylvania Institutional Review Board approval. This exemption may or may not be sufficient at your local institution. Should you have any questions or concerns, please feel free to reach out to us*”.

The Qualtrics platform (Provo, UT, USA; account license purchased by the University of Pennsylvania) was used to create an electronic multiple-choice survey for the current descriptive study. The questionnaire was developed based on the AMEE guideline No.87 (17) and the questions were designed to evaluate the academic curriculum and clinical protocols on the topic of OSA in postgraduate programs. In brief, a literature review on the topic of orthodontist's role in the management of OSA was conducted to ensure the proper background knowledge of the personnel who was developing the questionnaire. Utilizing the AAO White Paper as framework (4), the modules and survey items were developed. The survey first required respondents to indicate whether orthodontic management of OSA patients was part of the program curriculum. Then, a series of follow-up questions pertinent to the previous responses were prompted, to gauge the depth and scope of OSA-related training in both adult and pediatric populations as outlined in the AAO White Paper (4). Additional follow-up items examined the OSA-related expertise of educators in programs that included OSA curriculum, as well as the extent of training on identifying OSA signs and symptoms and the orthodontic management of OSA patients recommended by the AAO White Paper (4). Questions required the selection of one or multiple answer choices or an input of additional comments under the answer choice “other”. At the end of the survey, respondents were asked to indicate their confidence level in their residents’ ability to assess and manage OSA patients on a 5-point Likert scale. The draft of the questionnaire was sent to two orthodontic educators for feedback. Based on the feedback, the questionnaire was further modified, and several interviews were conducted with these two orthodontic educators to ensure that they understood the items as intended. A pilot survey was sent to three full-time orthodontic faculty members for feedback on survey completion time, clarification, and content validation. These three full-time orthodontic faculty members serve in the same institution, so the consistency among their responses was used to further validate the clarity of the current questionnaires.

For the formal study, anonymous survey links were sent directly from Qualtrics via email to study participants, consisting of the program directors of all 74 CODA (Commission on Dental Accreditation)-accredited postgraduate orthodontic programs in North America (obtained from the American Association of Orthodontists website: https://www2.aaoinfo.org/programs-for-residents-and-educators/accredited-orthodontic-programs/ in year 2024). Qualtrics generated a unique survey link for each participant, ensuring only one response per participant while maintaining anonymity by not including identifiable information. Following the initial survey distribution, reminder emails were sent to nonrespondents at two time points: after two weeks and one month of the initial email. Undeliverable emails (i.e., bounced or failed) were attempted to be redirected to the appropriate recipient, such as department chairs. Responses were collected from July to September 2024, and links remained active for two months after the final reminder email.

Once surveys were completed, all responses stored on Qualtrics were exported onto GraphPad Prism (version 8.2.1, San Diego, CA, USA) for further analysis, visualization, and figure generation. For the questions that only allow for only one answer to be selected, the data was analyzed with parts of whole analyses and displayed as fraction of total. For the questions that allow for more than one answer to be selected, the frequency of each answer being selected were displayed with bar graph.

## 3 Results

### 3.1 Delivery of training on OSA management

**Figure 1:**
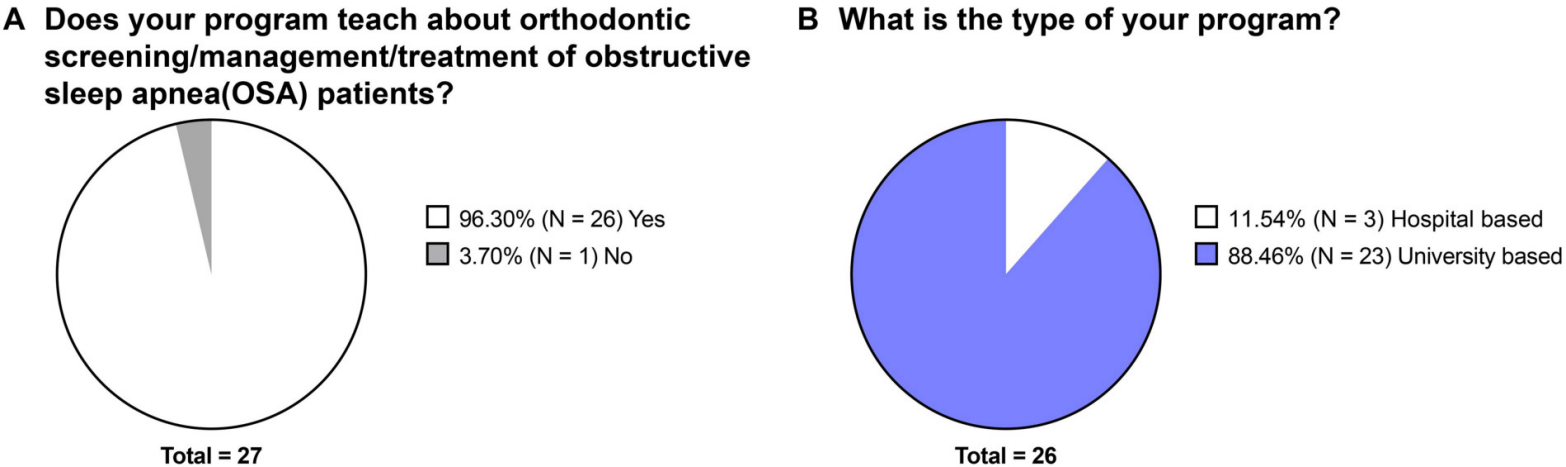
The overall information on whether the postgraduate orthodontic programs provide obstructive sleep apnea-related education. **(A)** Responses to the question “Does your program teach about orthodontic screening/management/treatment of obstructive sleep apnea patients?” **(B)** Responses to the question “What is the type of your program?”.

Twenty-seven program directors/department chairs completed the response to the survey with a response rate of 36.5%. Among them, 26 programs (3 hospital-based programs and 23 university-based programs) indicated that they provide training about orthodontic screening/management/treatment of OSA patients (Figure 1).

**Figure 2:**
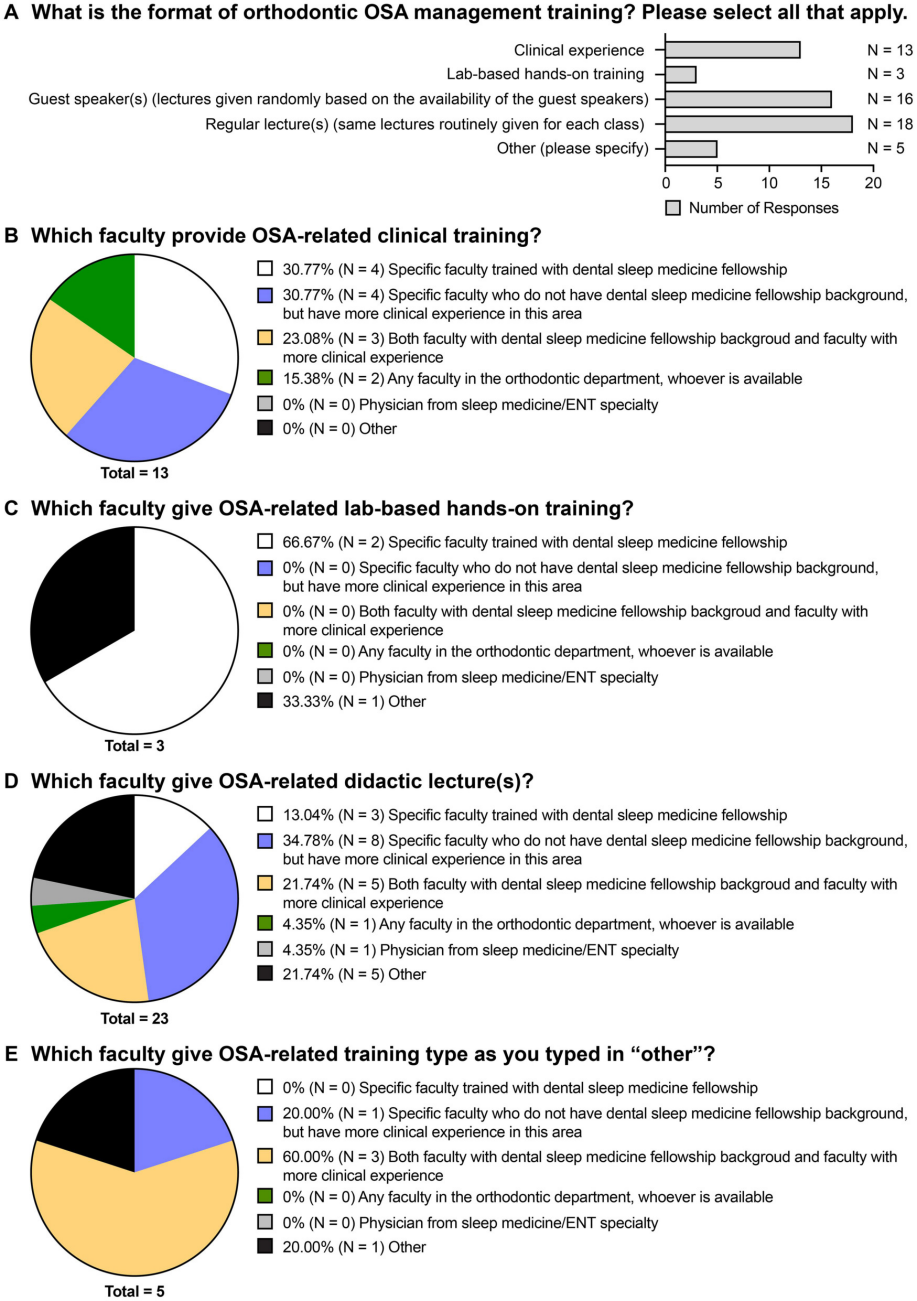
The format of training and the faculty resources provided by the orthodontic programs on the topic of obstructive sleep apnea. **(A)** Responses to the question “What is the format of orthodontic OSA management training? Please select all that apply.” **(B)** Responses to the question “Which faculty provide OSA-related clinical training?” **(C)** Responses to the question “Which faculty give OSA-related lab-based hands-on training?” **(D)** Responses to the question “Which faculty give OSA-related didactic lecture**(s)**?” **(E)** Responses to the question “Which faculty give OSA-related training type as you typed in ‘other’?”.

Regarding the format of OSA management training provided by these programs, routine lectures is the most common way (18 responses) (Figure 2A), with the hours of didactic lecture ranging from 2 hours per year to 5–10 h per month. The occasional lecture by guest speakers (16 responses) is the second most utilized format, followed by clinical experience (13 responses) (Figure 2A). Few programs provided lab-based hands-on training (3 responses) (Figure 2A). The other format of trainings indicated by respondents included: 1) week-long rotation in the first year through the sleep program at the hospital with the orthodontic residents learning/seeing/interpreting clinical examinations, home sleep studies, in lab sleep studies, follow up visits, etc.; 2) OSA related thesis or research project presentation to fellow residents; 3) interactive seminar; and 4) literature reviews.

When further elaborating on the faculty resources for each format of OSA management training, majority of the programs have specific faculty trained with dental sleep medicine fellowship and/or specific faculty who do not have sleep medicine fellowship background but have more clinical experience in this area as the predominant teaching personnel to provide each type of OSA training (Figures 2B–E). Other types of faculty who are involved in didactic teaching are oral medicine faculty, sleep physicians, prosthodontists with sleep medicine background, and pulmonologists. In addition, for the week-long rotation mentioned above, the teaching faculty are pulmonologists.

### 3.2 OSA screening protocol

**Figure 3:**
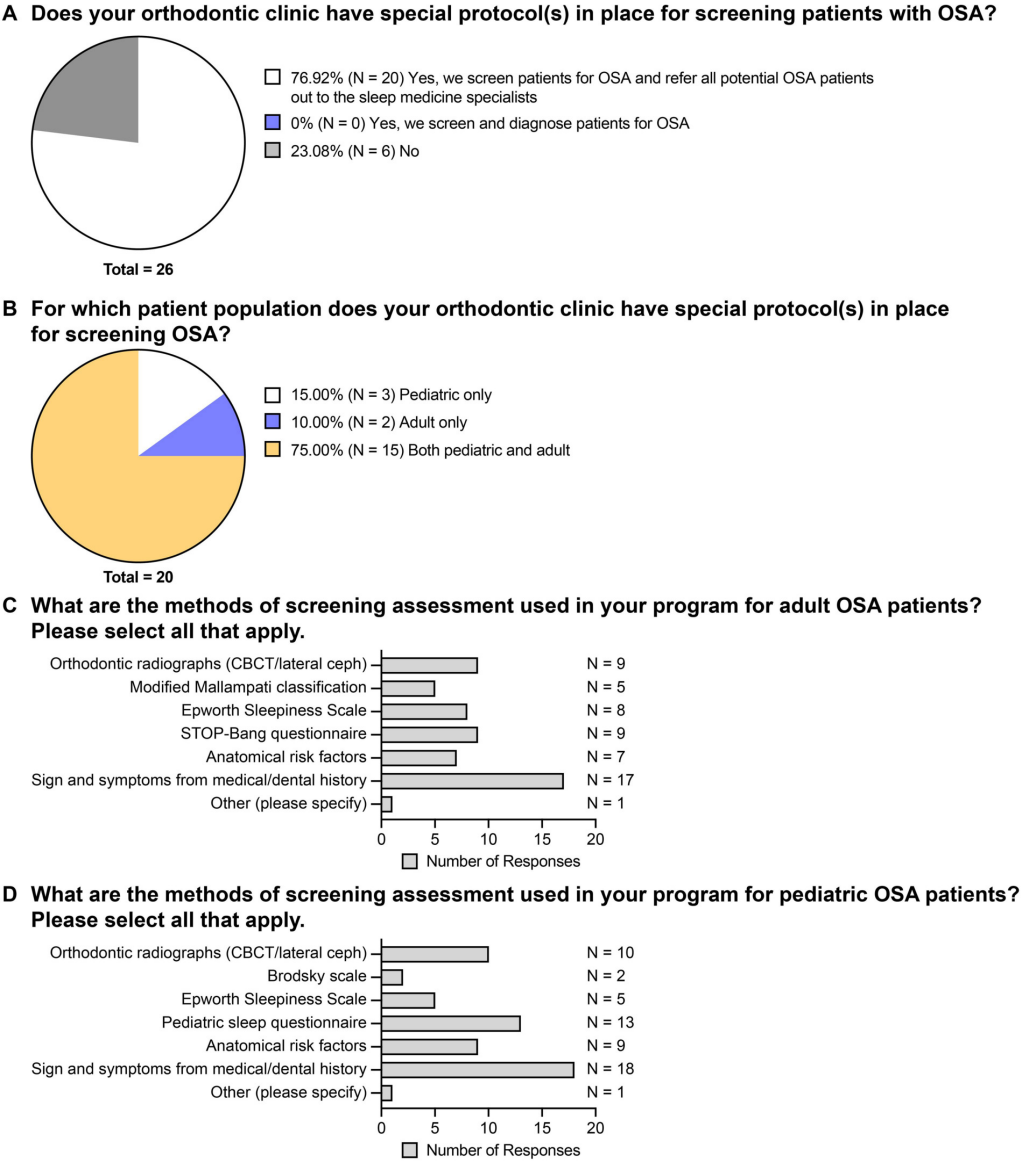
The availability of clinical protocol regarding screening patients with OSA. **(A)** Responses to the question “Does your orthodontic clinic have special protocol**(s)** in place for screening patients with OSA?” **(B)** Responses to the question “For which patient population does your orthodontic clinic have special protocol**(s)** in place for screening OSA?” **(C)** Responses to the question “What are the methods of screening assessment used in your program for adult OSA patients? Please select all that apply.” **(D)** Responses to the question “What are the methods of screening assessment used in your program for pediatric OSA patients? Please select all that apply.”.

In the post-graduate orthodontic clinic, 20 of the 26 responding programs indicated that they screen patients for OSA and refer all potential OSA patients out to the sleep medicine specialists, while the other six programs indicated that they do not have a specific protocol in place for screening patients with OSA (Figure 3A). Furthermore, for the programs that have OSA screening protocol(s), 75% of them have screening protocol for both pediatric and adult patient populations, 15% of them have screening protocol only for pediatric patients, and 10% have screening protocol only for adult patients (Figure 3B).

Screening assessment for adult OSA patients mostly relied on signs and symptoms from medical or dental history (17 responses) (Figure 3C). Other screening methods utilized include orthodontic radiographs such as CBCT and lateral cephalogram (9 responses), STOP-Bang questionnaire (9 responses), Epworth Sleepiness Scale (8 responses), anatomic risk factors (7 responses), and Modified Mallampati classification (5 responses) (Figure 3C). One program also stated that they utilized the NOSE (Nasal Obstruction and Septoplasty Effectiveness) score for adult OSA patient screening.

For pediatric patients, OSA screening assessment mostly relied on signs and symptoms from medical or dental history (18 responses) as well (Figure 3D). Other screening methods include pediatric sleep questionnaire (13 responses), orthodontic radiographs such as CBCT and lateral cephalogram (10 responses), anatomical risk factors (9 responses), Epworth Sleepiness scale (5 responses), and Brodsky scale (2 responses) (Figure 3D). The NOSE score is also utilized by one program for pediatric OSA screening.

### 3.3 OSA treatment protocol

**Figure 4:**
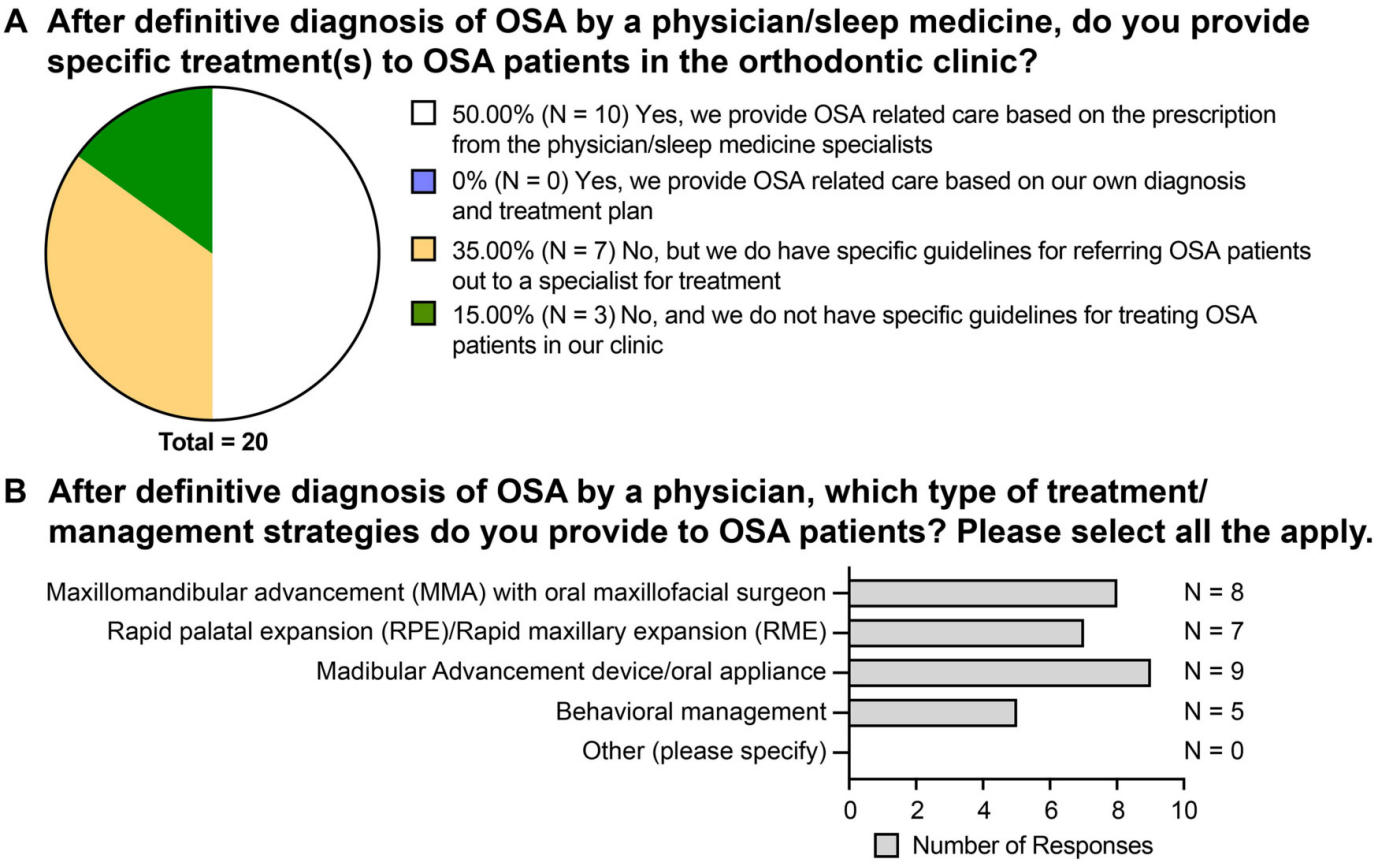
The OSA-related orthodontic care provided by the postgraduate orthodontic programs. **(A)** Responses to the question “After definitive diagnosis of OSA by a physician/sleep medicine, do you provide specific treatment**(s)** to OSA patients in the orthodontic clinic?” **(B)** Responses to the question “After definitive diagnosis of OSA by a physician, which type of treatment/management strategies do you provide to OSA patients? Please select all that apply.”.

In terms of treating patients diagnosed with OSA by a physician, 50% of orthodontic programs (10 responses) provided OSA-related care based on the prescription from physician or sleep medicine specialist, while 35% (7 responses) did not provide OSA-related care but had specific guidelines for referring OSA patients to a specialist for treatment (Figure 4A). Three programs (15%) did not provide OSA-related care nor have specific guidelines for treating OSA patients in orthodontic clinics (Figure 4A).

For the programs that do provide OSA-related care, mandibular advancement device or oral appliance (9 responses) is the most commonly provided type of care, followed by maxillomandibular advancement with oral surgeon (8 responses), rapid palatal expansion (7 responses), and behavioral management (5 responses) (Figure 4B).

### 3.4 OSA training outcome assessments

**Figure 5:**
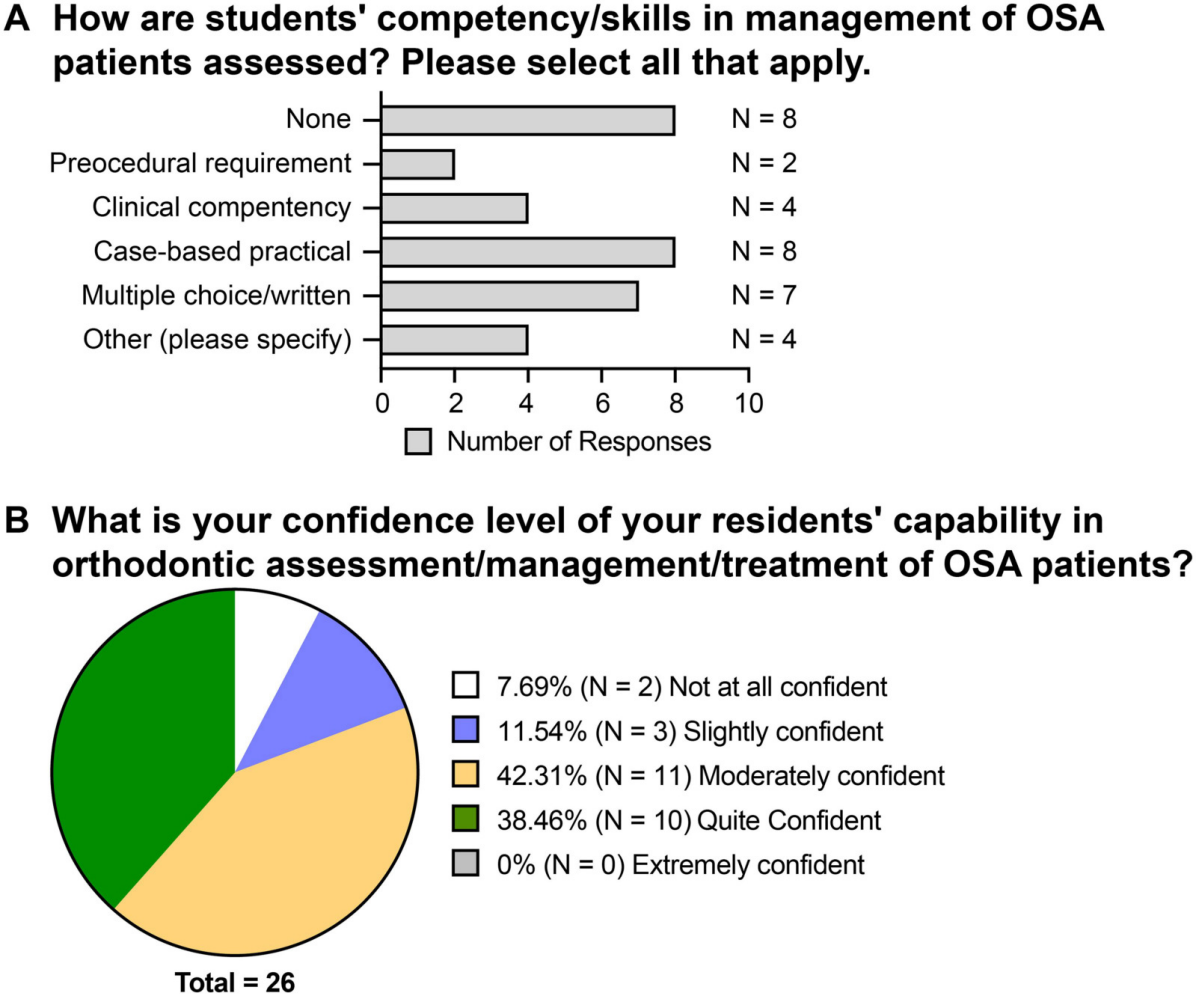
The teaching outcome assessment and the confidence level of the program directors for residents’ capability in OSA-related orthodontic care. **(A)** Responses to the question “How are students’ competency/skills in management of OSA patients assessed? Please select all that apply” **(B)** Responses to the question “What is your confidence level of your residents’ capability in orthodontic assessment/management/treatment/of OSA patients?”.

Lastly, the assessment of OSA training outcome has been investigated. Eight programs responded that they do not assess the students’ competency/skills in management of OSA patients (Figure 5A). For the programs that do assess, case-based practical is the most common method (8 responses), followed by multiple choice or written exams (7 responses), clinical competency (4 responses), and procedural requirement (2 responses) (Figure 5A). Other assessment formats that have been reported are oral assessment, completion of training, and conversations during case management follow-ups.

In regards to the program directors’ confidence level of their residents’ capability in orthodontic assessment/management/treatment of OSA patient, 38.46% (10 responses) reported being “quite confident” in their residents’ ability to assess, manage and treat OSA patients; 42.31% (11 responses) were “moderately confident”; 11.54% (3 responses) were “slightly confident”; and 7.69% (2 responses) were “not at all confident” (Figure 5B). No respondent reported being “extremely confident”.

## 4 Discussion

Obstructive sleep apnea has been a worldwide issue, with an estimated 936 million adults aged 30–69 years (men and women) having mild to severe OSA, and 425 million adults aged 30–69 years having moderate to severe OSA globally (18). However, the currently available literature demonstrated the total amount of teaching time spent on sleep medicine in undergraduate/pre-doctoral dental programs was limited with the range as an average of 1.2 h–5.6 h (10–14). And the only study on evaluating the curriculum of OSA-related training in post-graduate orthodontic residency was conducted in Thailand, and it reported an apparent absence of definitive guidelines for OSA-related curriculum (9, 15). It is worth noting that while several countries have high rates of OSA, the United States is one of the five countries with the highest OSA prevalence (18). Given orthodontists’ expertise in evaluating facial disharmony and the ongoing patient contact inherent to orthodontic treatment, they are well-positioned to screen, assess, and manage OSA (4, 19). Thus, fully understanding whether the current orthodontic education properly prepares the resident to provide care to the patients with OSA, especially for the North American region, is urgently needed.

In the current study, the majority of the responding post-graduate orthodontic residency programs stated that they provide OSA-related training to the residents (Figure 1A); however, the format of OSA-related education was primarily didactic, consisting of routine lectures and occasional guest speakers (Figure 2A), which is similar to the findings of Peanchitlertkajorn et al. regarding the OSA-related training in post-graduate orthodontic residency in Thailand (15). Although existing literature on orthodontic education has highlighted the importance of integrating theoretical knowledge and hands-on clinical training to prepare residents for complex clinical scenarios (20, 21), only half of the respondents reported clinical experience as part of their OSA training (Figure 2A). In addition, for the programs relying on didactic methods, there was a large variation regarding the type of faculty providing OSA-related lectures (Figure 2D). This inequality in faculty expertise and clinical exposure may hinder residents’ ability to develop the comprehensive skills necessary for OSA assessment and management in some residency programs.

In addition to the structure and delivery of OSA education, OSA screening protocols were also examined in this study. About 23% of respondents did not have a special protocol in place for screening patients with OSA (Figure 3A). According to the AAO White Paper, orthodontists must be familiar with the signs and symptoms of adult and pediatric OSA, as well as anatomical risk factors of airway obstruction, such as tongue position and tonsillar hypertrophy (4, 22). However, only 75% of respondents had specific screening protocols for both adult and pediatric patients, indicating they routinely screen for OSA (Figure 3A). Without standardized screening protocols for both adult and pediatric patients, residents may lack sufficient exposure to develop proper clinical experience in OSA assessment. In addition, almost all respondents who screened for OSA depended heavily on signs and symptoms from medical and dental history. Few respondents utilized OSA screening tools such as the STOP-Bang questionnaire and pediatric sleep questionnaire (Figures 3C,D) as the AAO White Paper recommended (4). Thus, it appears that current screening practices remain inconsistent and may fall short of established guidelines, potentially limiting residents’ preparedness to identify patients at a risk for OSA.

When encountering patients diagnosed with OSA, only 50% of respondents collaborated with the physician and provided OSA-related orthodontic care (Figure 4A). The limited interdisciplinary engagement between airway specialist and orthodontist within orthodontic residency programs is notable, given the critical role orthodontists can play in the comprehensive management of OSA. While orthodontic treatments with RME and mandibular advancement devices have been shown to increase oropharyngeal volume, their effects on oxygen saturation and OSA symptom relief remain controversial (23–25). The combination of limited OSA education in orthodontic programs and the uncertainty regarding the clinical efficacy of these orthodontic interventions highlights the need for further studies to focus on the effect of orthodontic treatment on airway function rather than on airway volume in order to improve the OSA-related curriculum of orthodontists.

With a large diversity in OSA teaching among programs, only 38.46% respondents reported being quite confident in their residents’ ability in assessment and management of OSA patients, and about 20% respondents were not confident or only slightly confident.

Nevertheless, it is important to note the limitations of the current study. Firstly, a new questionnaire was generated in the current study. Although the clarity of the questionnaire was validated by the consistency among the responses of three full-time orthodontic faculty members who serve in the same institution, a larger-scale construct validation with participants from different institutions is lacking. Secondly, the response rate was 36.5% (27 responses) and non-response bias could be a limitation. However, the distribution of hospital- vs. university-based programs in the sample (11.54% vs. 88.46%) is similar to that of the population (10.81% vs. 89.20%). Whether the hospital- and university-based programs differ in OSA training warrant further investigations. In addition, programs more engaged in OSA education may be more likely to respond, and the faculty expertise may influence program directors’ confidence. In other words, OSA curriculum may be even less consistent and orthodontic residents may be even less prepared in regard to OSA than the current study reports.

## 5 Conclusion

Our survey revealed a disconnection between the expectations placed on orthodontists and the training they receive in OSA screening and management. Attention from orthodontic educators is warranted to enhance the content, format, and delivery of training related to OSA to prepare future orthodontists to effectively identify and manage OSA patients within a strong collaboration with all involved specialists. Further studies with detailed quantifications and measurements on latent constructs, competencies, as well as comparisons among programs with different bases, at different locations, or with different patient populations would be beneficial to structure the standards of OSA curriculum in postgraduate orthodontic training.

## Data Availability

The original contributions presented in the study are included in the article/Supplementary Material, further inquiries can be directed to the corresponding author.
